# The use of pesticides in Polish agriculture after integrated pest management (IPM) implementation

**DOI:** 10.1007/s11356-020-12283-w

**Published:** 2021-01-25

**Authors:** Arkadiusz Piwowar

**Affiliations:** Wroclaw University of Economics and Business, Komandorska Street 118/120, 53-345 Wrocław, Poland

**Keywords:** Chemical plant protection, Integrated pest management (IPM), Multiple correspondence analysis (MPA)

## Abstract

The aim of the conducted study was to characterize the attitudes and practices of Polish farmers in the area of performing chemical plant protection treatments. A particular attention was paid to identifying the relationship between the direction of changes in the volume of chemical plant protection product consumption and selected attributes of farms. The main time range of the analyses covered the period of 2013–2017. Statistical data and results of representative surveys carried out on a sample of 1101 farms in Poland were used in the research process. Due to the large number of variants of the analysed variables, a multiple correspondence analysis was used, which made it possible to determine the correlation between the examined features (direction of changes in pesticide use relative to the farm area, economic size of the farm and location of the farm). Statistical analysis showed the existence of strong relationships between the physical (1) and economic (2) size of farms and the direction of changes in pesticide consumption ((1) *φ*^2^ = 0.0907; (2) *φ*^2^ = 0.1141)). According to empirical studies, the reduction of pesticide consumption took place mainly on the smallest farms. The implementation of the integrated plant protection directive has not resulted in significant changes in the form of reduced pesticide use in large-scale field crops. This raises the need to modify the strategy and model of crop protection in large-scale field crops in Poland.

## Introduction

Plant protection is an interdisciplinary field of knowledge, research and economic practice. Chemical plant protection is an integral part of effective, conventional and integrated farming (Aktar et al. 2009; Leong et al. 2020). This is because pesticides make it possible to increase crop yields by reducing losses due to plant diseases, foraging insects that feed on these plants, etc. The popularity of pesticide use in farming is due to the fact that chemical plant protection is relatively cheap and effective (Lechenet et al. 2017; Bernhardt et al. 2017; Hedlund et al. 2020). Much is expected after the development of biological methods in the field of plant protection, but still the effectiveness of most bio-pesticides is much lower than that of chemicals (Compant et al. 2005). Moreover, the expensive registration procedure and the more difficult and troublesome application of bio-pesticides do not support the dynamics of changes on the global market of plant protection products (Pavela and Benelli 2016; Bruce et al. 2017; Kerr and Bullard 2020; Isman 2020).

On the other hand, excessive use of pesticides is connected with environmental and health risks and also leads to the development of resistance to pesticides (Schäfer et al. 2007; Jallow et al. 2017; Fletcher et al. 2020; Serrano et al. 2020). The introduction of chemical protection agents into agrocenoses violates the ecological stability of the environment and has a direct impact on the biodiversity of ecosystems (Pimentel et al. 1993; Gagic et al. 2017; Klich et al. 2020). The chemical methods of plant protection have a negative effect on earthworm populations (Bart et al. 2018; Treder et al. 2020). It should be emphasized, however, that the most harmful products are systematically withdrawn from use (Lamichhane et al. 2016). Currently produced agrochemicals, including plant protection products, are innovative preparations in all aspects. Research and development works are constantly being carried out on optimizing the effectiveness and selectivity of the preparations and minimizing the impact of pesticide application on the health and safety of humans and animals (Liu and Huang 2013; Kumar et al. 2019; Mancini et al. 2019). An important direction of the research are pro-ecological technologies and techniques for the production of plant protection products based on immobilization processes, using bio-renewable resources, etc. (Włodarczyk and Siwek 2017; Ndao et al. 2020). Progress in toxicology, chemistry and biotechnology is being used (Chhipa 2017; Hazra and Purkait 2019; Slattery et al. 2019). Despite this, there is still discussion about the harmfulness of, e.g. one of the most popular herbicides in the world, whose active substance is glyphosate ([N-(phosphonomethyl) glycine]) (Silva et al. 2018; Myers et al. 2016; Benbrook 2019; Padilla and Selim 2020; Beckie et al. 2020; Kudsk and Mathiassen 2020; Rogacz et al. 2020). Generally, a lot of original publications by authors from various scientific and research centres as well as literature reviews indicate a relationship between the use of plant protection products and the increased risk of developmental disorders, cancer diseases, etc. (Tayour et al. 2019; Huang et al. 2019; Han et al. 2019; Sabarwal et al. 2018; Zeng et al. 2017; Narayan et al. 2017; Egbuna et al. 2020). Many papers also concern the safety of performing chemical plant protection treatments, including operator safety (Matthews 2008; Feola and Binder 2010; Damalas and Abdollahzadeh 2016). Declines in the number and diversity of insects have focused attention on the role of pesticides and their impact on health, including honeybees (Zawislak et al. 2019; Prado et al. 2019; Uhl and Brühl 2019; Crenna et al. 2020). A lot of studies show that bees are exposed to pesticides, although there are differences in sensitivity between bee species (Hladik et al. 2016; Woodcock et al. 2017; Arena and Sgolastra 2014). There is also the problem of residues and secondary emissions of pesticides with historical use (including DDT) (Cui et al. 2020). Nevertheless, despite the incentives introduced in many countries to reduce pesticide use and the transition to organic farming (e.g. Denmark, France, Germany, Spain, the Netherlands, the United Kingdom), there is no spectacular reduction in their use (Jørgensen et al. 2019; Chèze et al. 2020). The analyses performed by Hedlund et al. (2020) show the links between economic development and pesticide consumption over time, without reducing their use at higher levels of economic development. There is also a problem with pesticides in processed foods, including detection and elimination methods (Azam et al. 2020; Albaseer 2019; Wang et al. 2019; Fortunati et al. 2019; Czaja et al. 2020).

“Pesticide toxicity risk” is one of the most important indicators of the European Union’s (EU) agricultural policy, which is crucial for achieving the Sustainable Development Goals (SDGs) (Scown and Nicholas 2020). Due to threats to human health and the environment, agro-ecological approaches and new pest control strategies based on biological methods should be developed (Harrison et al. 2019; Southon et al. 2019; Yan et al. 2020; Lopes et al. 2016). It is also important to integrate mechanical care treatments with chemicals (herbicides, insecticides, etc.) (Zarzecka et al. 2020). Water pollution by pesticides used in agriculture is currently a major concern in Europe (Silva et al. 2019; Herrero-Hernández et al. 2020). Support is needed in the form of systems supporting farmers’ decisions. The flow of knowledge by passing on the results of interdisciplinary research in the field of natural and social sciences is significant. These studies may be aimed at assessing risk, economic efficiency, etc. (Vasileiadis et al. 2013; Lavik et al. 2000b).

Integrated pest management (IPM) is the point of reference for the analyses of pesticide consumption carried out in this study. The basis of this concept is a holistic approach to pest and plant disease control using all available methods, while minimizing the use of chemical pesticides (Barzman et al. 2015). In this way, the pressure on the natural environment is reduced, and the biodiversity of the agricultural environment is protected. As Kogan (1998) points out, the history of IPM goes back to the end of the nineteenth century, when ecology was recognized as the basis of scientific plant protection. Over the last several decades, there has been a methodological and organizational progress in the studied subject matter, which is developing methodologies including monitoring the occurrence of harmful organisms, determining the thresholds of their economic harmfulness, organizing training courses, thematic conferences and developing consultancy and decision support systems in plant protection (Ma and Abdulai 2019; Mondino and González-Andújar 2019; Dara 2019). IPM is an extremely important element in the pursuit of sustainable agriculture, based on deep knowledge of the agricultural environment, which is to ensure the production of high-quality food without degrading the agricultural environment. IPM is globally endorsed as the future paradigm for crop protection (Stenberg 2017). Contemporary challenges in the field of IPM are also important. The literature on the subject emphasizes the need to formulate general principles of synergistic combination of traditional and innovative measures in the field of IPM, including public and private policy instruments in the field of reducing pesticide use (Lamichhane et al. 2016; Stenberg 2017; Lee et al. 2019). Interesting challenges in the area of research are the motivations and actions of farmers. Moss (2019) points to, inter alia, identifying the reasons why farmers are reluctant to use non-chemical alternatives.

The spatial scope of analyses in this paper concerns Poland. Farmers in Poland make independent decisions regarding agrotechnical operations (type of technology and technique), and their behaviour in this respect must take into consideration the principles of environmental protection. The regulations in this area include the code of good agricultural practice (GAP), and compliance with the rules contained therein helps to apply plant protection products safely and effectively. Since January 1, 2014, farmers in Poland have been obliged to comply with the IPM principle, which imposed new rules on them. This is defined by numerous legal acts, in particular Directive 2009/128/EC of the European Parliament and of the Council (DUUE 2009) establishing a framework for community action on the sustainable use of pesticides (Lefebvre et al. 2015; Stenberg 2017). The rule is to use all available methods and techniques, especially non-chemical ones (Karlsson Green et al. 2020). The legal obligation to apply the general principles of IPM in Poland refers to all professional users of plant protection products.

Environmental degradation caused by the use of chemical plant protection products in cultivation contributes to the search not only for alternative methods, techniques and plant protection products, but also for changes in social and economic regulations. Currently, in Polish agriculture, there is a transition phase between conventional and sustainable agriculture (Niewiadomska et al. 2020). In this context, the behaviour of agricultural producers regarding the use of chemical means of agricultural production is extremely interesting (Damalas and Koutroubas 2018). According to many authors, research in this area is neglected. In recent years, research in the subject literature has relatively rarely undertaken behavioural studies in the context described in this paper among European farmers. Chèze et al. (2020) have used the method of discrete choice experiment that includes the risk of large production losses due to pests. The authors analysed farmers’ readiness to reduce the use of pesticides in France. These studies took into account, among others the following factors, the administrative framework of the practice change, the resulting reduction of the impact of pesticides on health and the environment and the risk of large production losses. The authors of these studies conclude that more research is needed to understand the drivers behind the adoption of pesticide reduction practices, especially local research. In turn, Lavik et al. (2020a) have analysed pest management strategies among Norwegian farmers (have used the method of multi-attribute decision analysis). These analyses show that the adoption of the IPM strategy can be accelerated through the granting of subsidies to farmers and the development of advisory services. A lot of research in the field of knowledge, attitudes and practices concerns African and Asian countries (Mekonnen and Agonafir 2002; Jallow et al. 2017; Mengistie et al. 2017).

The presented research is to fill the existing gap in the area of sustainable use of pesticides in Central and Eastern Europe. The use of pesticides in this area is a significant threat in terms of water and soil pollution (Székács et al. 2015; Hvězdová et al. 2018; Vašíčková et al. 2019). The aim of the study was to characterize the attitudes and practices of Polish farmers in the field of performing chemical plant protection treatments. A particular attention was paid to identifying the relationship between trends of changes in the volume of chemical plant protection products consumption and the farm area, its economic size and location (place). The main time range of the analyses covered the period of 2013–2017. In a broader context, the research aimed to assess the attitudes and practices of farmers in Poland in the field of sustainable agriculture and sustainability of modern agri-food markets and their development (Borsellino et al. 2020).

## Materials and methods

**Fig. 1.**
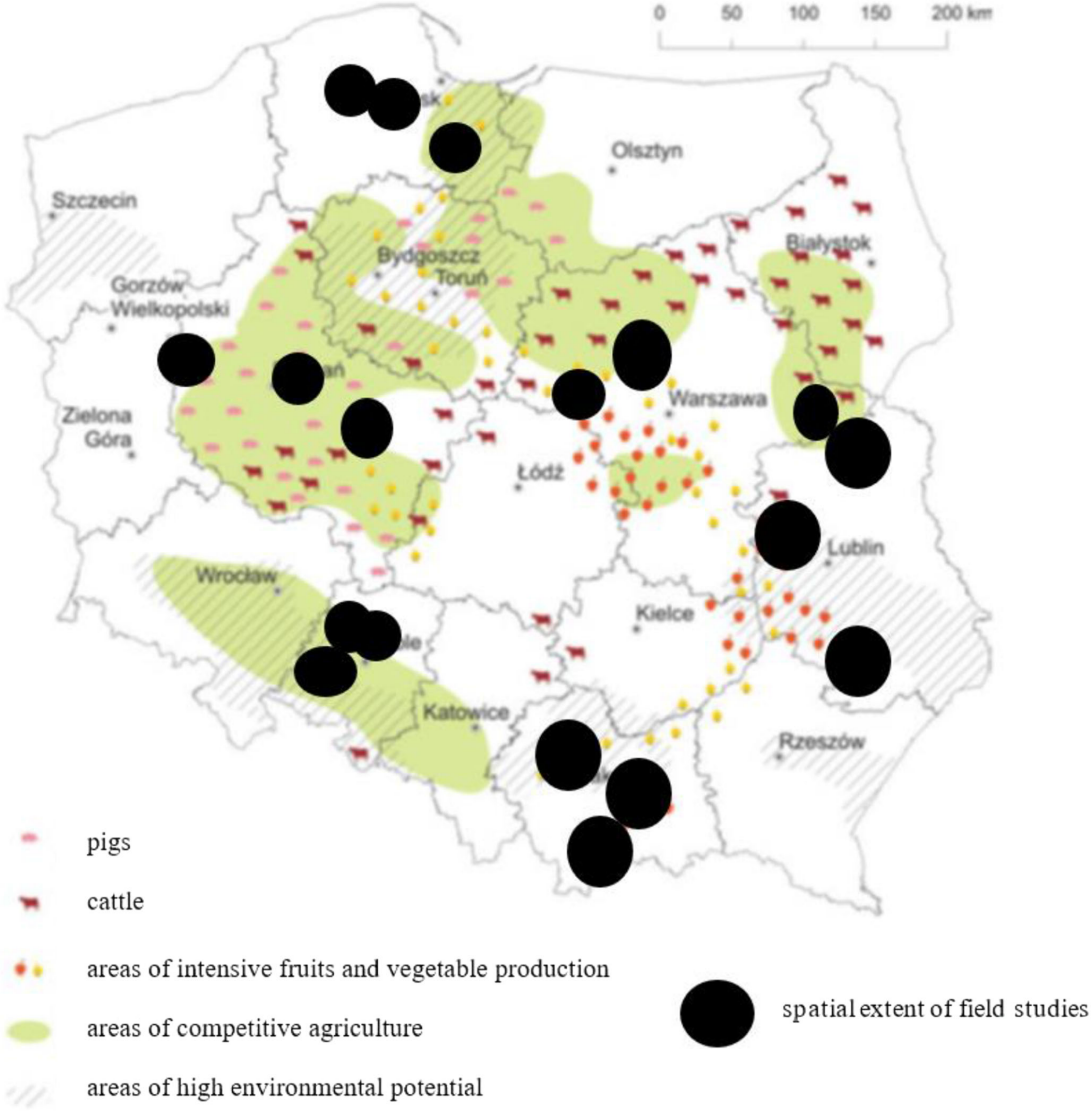
Main food producing areas in Poland and spatial extent of field studies. Source: Own elaboration based on https://www.igipz.pan.pl/tl_files/igipz/ZGWiRL/ARP/01.Znaczenie%20rolnictwa%20w%20gospodarce%20Polski.pdf (accessed 13.07.2020)

Achievement of the purpose of the work was related to collecting secondary and primary data. Statistical data on total pesticide consumption in Polish agriculture and in selected crops was collected and analysed. Due to the lack of information from secondary sources regarding the behaviour of farmers, which would allow the full achievement of the objectives of the work, a representative primary (survey) research was carried out among agricultural producers in November 2017–March 2018. Surveys were conducted in 18 districts in Poland. The selection of districts was random, layered. The first layer of the draw was constituted by provinces (one province was drawn from each of the six macro-regions in Poland). Then, three districts were drawn in each previously drawn province, which allowed field studies to be carried out in a diverse spatial, social and economic environment (Fig. 1).

In total, surveys were carried out in 1101 farms in Poland. Participation in the research was on a voluntary basis. The characteristics of respondents are presented in Table 1.

**Table 1:** Basic general characteristics of interviewed farmers

Specification	Population	Share in the test sample
	(pcs)	(%)
Age of respondents		
18–29 y	128	11.6
30–39 y	250	22.7
40–49 y	328	29.8
50–59 y	281	25.5
> 60 y	102	9.3
No data	12	1.1
Total	1101	100
Gender of respondents		
Women	197	17.9
Men	901	81.8
No data	3	0.3
Total	1101	100
Education level of respondents		
Primary	44	4.0
Graduate vocational school	389	35.3
Secondary	518	47.0
Higher	142	12.9
No data	8	0.7
Total	1101	100
Number of years worked in agricultural holding		
1–5 y	90	8.2
6–10 y	146	13.3
11–15 y	119	10.8
16–20 y	172	15.6
21–25 y	138	12.5
26–30 y	152	13.8
> 31 y	282	25.6
No data	2	0.2
Total	1101	100
Selected features of agricultural holdings		
Area of agricultural lands (ha)		
< 5	88	8.0
5–9.99	195	17.7
10–14.99	191	17.3
15–19.99	136	12.4
20–29.99	164	14.9
30–49.99	170	15.4
50–99.99	115	10.4
>100	41	3.7
No data	1	0.1
Total	1101	100
Economic size of agricultural holding (SO)		
€< 10,000	316	28.7
€10,100–13,000	156	14.2
€13,100–20,000	188	17.1
€20,100–50,000	232	21.1
€50,100–100,000	99	9.0
€100,100–200,000	40	3.6
€> 200,000	4	0.4
No data	66	6.0
Total	1101	100

Source: Own study based on questionnaire surveys (*N* = 1101)

The basic research tool was a survey questionnaire. The implementation of such extensive research took place in cooperation with agricultural advisory centres operating in the studied area. The data was collected through direct interviews conducted by an interviewer (agricultural advisor). In the survey, respondents indicated the direction of changes in the amount of consumed pesticides and described agricultural practices that are characteristic in the use of pesticides on their farm. The questions in the survey concerned the activities of agricultural producers in the period of 2013-2017. These practices are directly related to plant protection treatments and are listed in the label instruction. The respondents answered four questions (three variants of answers were possible: “yes,” “no,” “I don’t know”):

- I choose plant protection products in such a way as to minimize the negative impact of plant protection treatments on non-target organisms;
- I limit the number of treatments and the amount of used plant protection products to the necessary minimum;
- I counteract the emergence of resistance of harmful organisms to plant protection products through proper selection and alternate use of these products;
- I am familiar with the methodology of integrated plant management for individual crops on my farm.

Due to the fact that the research intention was to learn the coexistence of the direction of changes in the area of pesticide use with selected features of a farm, a multiple correspondence analysis was used. This method was successfully used in the same subject matter (assessment of environmental risk and the effectiveness of agrotechnical operations, farmers’ behaviour regarding the use of plant protection products, fertilizers, etc.) (Dmowska and Ilieva 1995; Piwowar 2020; Svobodová et al. 2020). The input data was compiled in the form of a Burt table (symmetrical block matrix). Then the percentage distributions in rows and columns, total distributions, expected numbers, differences between observed and expected numbers, standardized deviations and share in the total value of chi-square statistics were determined. The analysis of correspondence made in the paper was carried out using the computer program STATISTICA 13.3. Research methods of descriptive statistics and analysis were also used, with particular reference to comparative and literature analysis and descriptive methods. The analyses carried out in this work are a section of the research area related to low-carbon agriculture development in Poland. The work related to this research project was financed by the National Science Centre in Poland.

### The use of pesticides in Polish agriculture—volume and structure of consumption

**Fig. 2.**
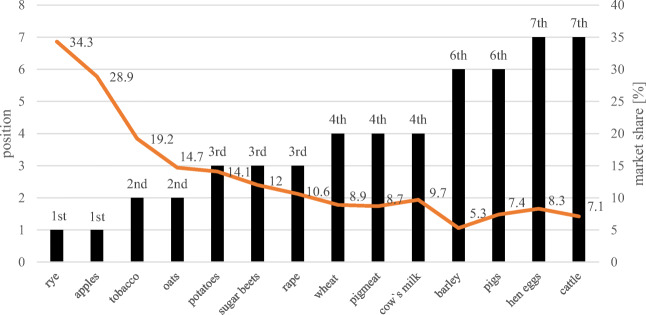
Market share and position of Poland in the classification in terms of the production volume of selected agricultural products in the European Union in 2018. Source: Statistical Yearbook of Agriculture, Statistics Poland, Warsaw 2020

Poland is a leading food producer in the European Union (Firlej et al. 2017; Bojnec and Fertő 2019). Taking into consideration agricultural production, Poland is at the forefront in the production of poultry meat, and in the field of plant production—wheat, rapeseed, sugar beet, triticale and rye. Poland is also distinguished by a high volume of berry fruit production (mainly strawberries, raspberries and currants), as well as being one of the largest producers of onions, cabbage and cauliflowers (Fig. 2).

Agricultural production in Poland in 2018 covered an area of 18.608 million ha. Due to the share of agricultural area in the total area, Poland is in third place in Europe (behind France and Spain). A characteristic feature of Polish agriculture is the large diversity and fragmentation of farms in terms of area. The average usable agricultural area (UAA) per farm in 2018 in Poland was 11.3 ha. The average farm size has been increasing in recent years (in 2002, it was 5.8 ha; in 2011, 9.1 ha) (Rolnictwo 2019). Despite the increase in the average area of a farm, still more than half of farms in Poland (51.9%) operate on no more than 5 ha of UAA. However, these farms have only 12.8% of total utilized agricultural areas in Poland (Rolnictwo 2019).

In the European Union countries, there are clear differences in the volume of pesticide sales, which depends on the natural conditions of agricultural production in a particular area. Taking into consideration the individual groups of plant protection products, compared with the countries of Western and Southern Europe, relatively large amounts of herbicides are used in Poland (over 50% of sales structure) (Table 2).

**Table 2:** Sales of pesticides in EU in 2018

Specification	Fungicides and bacteriocides	Herbicides, haulm destructors and moss killers	Insecticides and acaricides	Molluscicides	Plant growth regulators	Other plant protection products
	(tonnes)					
Belgium	2458	2648	476	16	269	769
Bulgaria	1798	2607	593	(c)	18	25
Czechia	1755	2572	292	8	265	287
Denmark	484	1905	44	15	202	1
Germany	11,682	14,533	16,237	154	2138	181
Estonia	107	428	29	(c)	73	(c)
Ireland	602	1833	29	10	161	17
Greece	1729	1833	1009	2	119	169
Spain	38,067	16,593	6488	(c)	195	(c)
France	39,087	34,392	5728	385	3567	1811
Croatia	767	718	127	2	80	4
Italy	31,539	6880	1653	36	475	13,455
Cyprus	823	161	151	2	0	47
Latvia	213	965	36	5	355	13
Lithuania	677	1054	57	(c)	262	(c)
Luxembourg	(c)	54	(c)	0	8	(c)
Hungary	3535	3824	787	1	169	219
Malta	83	3	3	1	0	(c)
Netherlands	4288	2978	243	11	386	1476
Austria	2269	1277	1569	6	84	75
Poland	7992	11,371	1770	(c)	1609	415
Portugal	4335	1939	675	11	3	1095
Romania	4542	5188	1012	5	313	48
Slovenia	849	257	55	2	5	4
Slovakia	676	1329	151	1	268	65
Finland	3814	982	21	1	68	14
Sweden	223	1483	94	(c)	50	22

Source: https://ec.europa.eu/eurostat/statistics-explained/images/b/be/Table_1_Sales_of_pesticides%2C_by_country%2C_2011_and_2018_%28tonnes%29.png (accessed 24.09.2020)

*(c)* confidential value

**Fig. 3.**
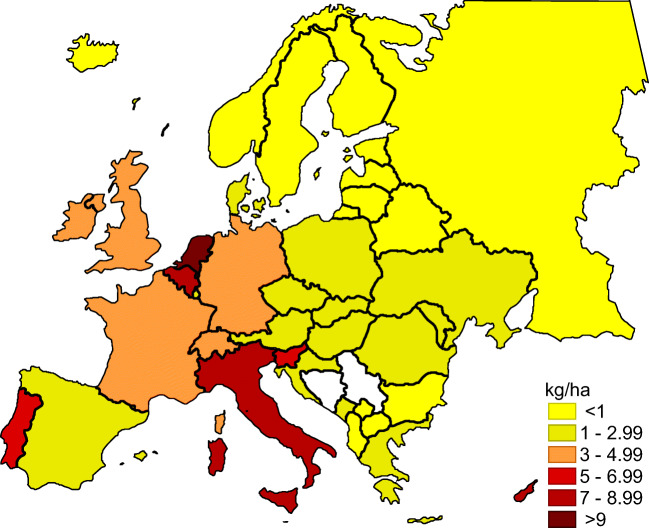
Average use of pesticides per area of cropland from 2014 to 2018 in European countries. Source: Authors’ own elaboration based on http://www.fao.org/faostat/en/#data/EP/visualize (accessed 14.10.2020)

Currently, it should be stated that the level of pesticide consumption is actually relatively low in relation to other European Union countries (Fig. 3).

**Fig. 4.**
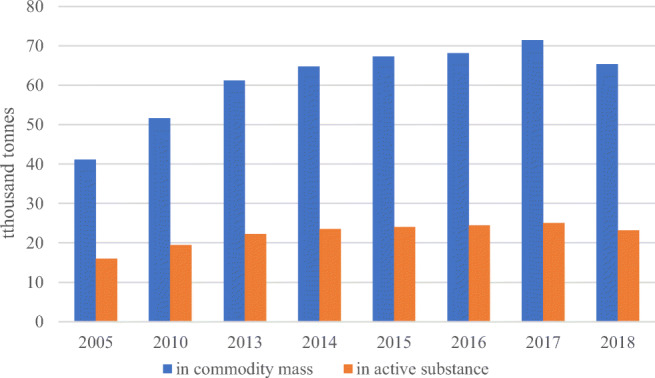
Sales of plant protection products in Poland. Deliveries on the domestic market by producers and importers; from 2018 by holders of the authorization of the Minister of Agriculture and Rural Development for the marketing of plant protection products. Source: Authors’ own elaboration based on Means of production in agriculture in the 2018/19 farming year. Statistics Poland, Warsaw 2020

According to http://www.fao.org/faostat/en/#data/EP/visualize (accessed 14.10.2020) data, the average pesticide consumption in Poland in 2014–2018 was 2.12 kg/ha. Higher consumption was recorded in each of the Western European countries, including Germany (3.9 kg/ha), France (3.83 kg/ha) and Spain (3.57 kg/ha). An important issue in the studied problems is, to a large extent, the lack of information regarding the volume of pesticide use in spatial terms. Previous practices in official statistics in this area do not meet the analytical needs of researchers of this phenomenon for the needs of practice, science and politics. Data on the overall volume of sales of plant protection products in Poland are presented in Fig. 4. In order to present a broader context in the area of pesticide sales, the time range of analyses was extended by the years 2005, 2010 and 2013–2018.

The volume of sales of plant protection products in Poland in 2005–2018 increased by 24,200 tonnes (by mass) and in 2018 amounted to 65,335 tonnes. In terms of tonnage, expressed in the amount of active substance, the increase in pesticide sales in Poland in 2005–2018 amounted to 7139 tonnes. It is worth noting that the volume of sales of plant protection products in Poland was increasing steadily until 2017. A decrease in dynamics and a reversal of trends were recorded in 2018. In 2018, the volume of sales of pesticides in Poland expressed in the amount of active substance (23,178 tonnes) was slightly lower than in 2014 (23,557 tonnes).

Considering the generic structure, herbicides are consumed the most in Poland. In 2018, sales of this product group (in terms of active substance) amounted to 11,370.7 tonnes (Table 2). The group of “Herbicides, haulm destructors and moss killers” constituted half of the pesticides sold on the Polish market. In the sales structure of pesticides, in addition to herbicides, in terms of quantity, an important group of plant protection products were fungicidal and bactericidal preparations (34.5%).

Data from official statistics in Poland (Rynek 2019) regarding the scope of applied chemical protection relate to the consumption of chemical agents in the cultivation of a few crop species (Table 3).

**Table 3:** Consumption of plant protection products in the studied crops in Poland in 2014–2018 (in kg of active substance/ha of crop)

Specification	Fungicides and bactericides	Herbicides, haulm destructors and moss killers	Insecticides and acaricides	Other plant protection products	Total
2014					
Fodder beet	0.06	0.81	0.01	0.00	0.88
Spring wheat	0.21	0.38	0.00	0.09	0.68
Oat	0.01	0.52	0.00	0.01	0.54
Currant	1.02	0.59	0.10	0.00	1.71
Plum	1.68	0.37	0.32	0.00	2.37
2015					
Cereal mixtures	0.01	0.55	0.00	0.00	0.56
Winter barley	0.45	0.60	0.01	0.01	1.12
Cherries	2.07	0.01	0.06	0.00	2.14
2016					
Winter tricale	0.24	0.44	0.01	0.08	0.76
Corn	0.02	0.71	0.01	0.01	0.75
Sugar beet	0.25	2.35	0.04	0.03	2.67
Onion	2.46	1.78	0.02	0.30	4.56
Carrot	0.55	0.96	0.15	0.00	1.66
Pear	5.61	0.13	0.34	0.00	6.08
2017					
Cucumbers	3.80	0.04	0.01	0.00	3.85
Cucumber under shelter	2.93	0.00	0.04	0.00	2.98
Ground tomatoes	6.69	0.36	0.00	0.19	7.24
Tomatoes under shelter	2.53	0.00	0.06	0.00	2.60
Winter wheat	0.55	0.41	0.05	0.31	1.32
Strawberries	1.95	0.73	0.04	0.03	2.74
Potatoes	2.79	0.64	0.04	0.01	3.49
Rye	0.08	0.17	0.00	0.06	0.31
2018					
Apple	9.90	0.30	0.25	0.01	10.46
Spring barley	0.11	0.48	0.01	0.02	0.62
Raspberries	1.08	0.21	0.04	0.00	1.33
Winter rape	0.45	0.92	0.29	0.08	1.74
Sour cherry	5.28	0.28	0.05	0.00	5.60

Source: Authors’ own elaboration based on *Rynek środków produkcji dla rolnictwa*. Analizy Rynkowe 2019, nr 46, p. 25 [In Polish]

Both presented data in Table 3 as well as the literature (Łozowska et al. 2013; Jankowska et al. 2019; Szpyrka et al. 2016) on the subject prove that high consumption of pesticides is particularly noted in horticultural production (e.g. apples and pears). In Poland, due to the high share of cereals in arable land and the significant importance of gardening, herbicides and fungicides are used most. Insecticides and acaricides are much less used.

### Pesticide use practices in Poland—results of empirical research

As mentioned in the introduction, IPM has been in force in Poland since January 1, 2014. Its main assumption is the priority of using non-chemical methods of plant protection. One of the basic principles of plant protection at the farm level is the rational use of plant protection products. In order to make it happen, it is necessary to be familiar with the current methodologies of integrated plant protection, plant protection programs and indications for signalling harmful organisms and implement decision support systems in plant protection (Swiergiel et al. 2019; Szeląg-Sikora et al. 2019). In relation to the thematic scope of the paper, i.e. indicating practices in the field of chemical plant protection, below are the respondents’ answers to four key issues.

**Fig. 5.**
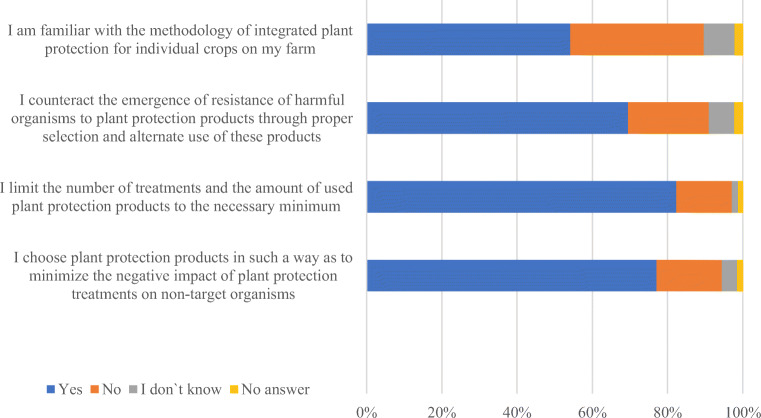
Respondents’ declarations regarding practical aspects of performing plant protection treatments. Source: Own study based on questionnaire surveys (*N* = 1101)

According to the research, more than half of the respondents declared knowledge of integrated plant protection methodology, counteracted the emergence of resistance of harmful organisms, limited the number of treatments and selected appropriate pesticides (Fig. 5).

The analyses show that the majority of respondents (82.4%) declared to minimize the number of treatments and the amount of plant protection products. However, the declared knowledge of the integrated protection methodology is worrying. This is due to the fact that 35.5% of the respondents declared they did not know the methodology of IPM for individual crops on their farm. This raises questions about the effectiveness of training activities, including mandatory training for people who perform plant protection treatments in agriculture. The certificate of completion of such training is valid for a period of 5 years from the date of completion of the training and entitles to perform treatments using plant protection products.

Inspired by the results obtained concerning the change in the amount of pesticide consumption declared by farmers, a study was carried out on the relationship between the direction of change and the characteristics (attributes) of a farm. Four variables were selected for detailed analyses: (1) direction of changes in the amount of pesticide use, (2) area of arable land, (3) economic size of a farm and (4) district. In order to determine the existence of dependence and its strength, values of χ^2^ statistics and mean square multivariate were calculated (Table 4).

**Table 4:** Statistics values χ^2^, critical values χ^2^_α = 0.01_* (in parentheses) and mean square contingency *φ*^2^ (italics) for the following features: pesticide use, arable land area, economics size of agriculture holding, and district

Specification	Arable land area *c* = 5**	Economic size of agricultural holding *c* = 6**	District *c* = 6**
Pesticide use *r* = 3**	93.8249 (20.0902) *0.0907*	117.9304 (23.2093) *0.1141*	49.5906 (23.2093) *0.0480*

Source: Own study based on questionnaire surveys (*N* = 1101)

* Critical values χ^2^_α_ = 0.01 read from the tables for (*r* − 1) × *(c* − 1) degrees of freedom

**Number of rows and columns of the variables analysed

The critical values read from the chi-square distribution tables, at a given level of significance, for all pairs of features (**χ**^**2**^_**α**_ = 20.0902 or 23.2093), are smaller than the calculated χ^2^ statistics (93.8249, arable land area; 117.9304, economic size; 49.5906, district). This means that the hypothesis about the independence of the examined features should be rejected, thus stating that the direction of changes in the amount of pesticide consumption depends on the area of the farm and its economic size and the district in which the farm is located.

**Fig. 6:**
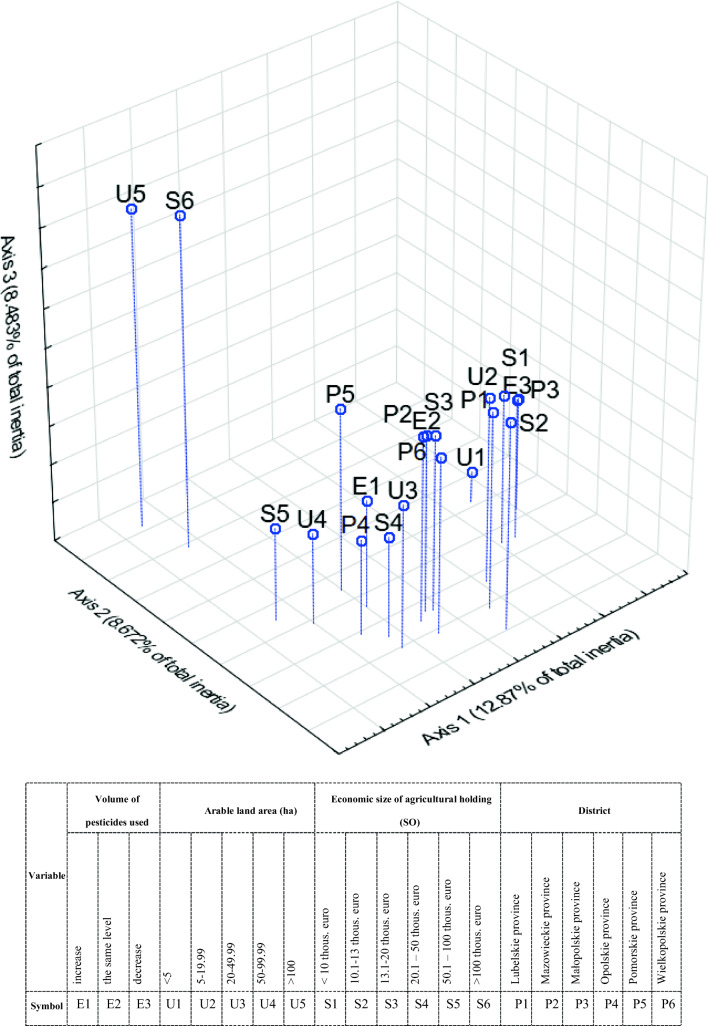
Graphic presentation of the results of the correspondence analysis of the direction of changes in the level of pesticides use with the analysed features of agricultural holdings. Source: Own study based on questionnaire surveys (*N* = 1101)

Figure 6 presents in three-dimensional space the result of an analysis of correspondence of the declared direction of changes in pesticide consumption (from the period of 2013–2017) with the features of farms. The use of correspondence analysis allowed presenting graphically the relationships among the studied variables.

Correspondence analysis showed that there is a coexistence between variable E3 (decrease in pesticide consumption) and variables U1 (farms below 5 ha of UAA) and S1 (economic size below €10,000). Moreover, agricultural producers, who declared an increase in pesticide consumption (E1), ran relatively larger farms, which are economically stronger (U4, S5). The correlation between declarations of increase in pesticide use and districts in which intensive agricultural production is carried out was also characteristic (P4, P5). The location of point E2 (no change in the amount of pesticide consumption) indicates average profiles in terms of the studied variables. This arrangement of points indicates that there is a slight differentiation between these categories.

The problem of linking farm characteristics with the direction of changes in agrochemicals consumption is an interesting research area. According to the author, a feature of modern plant protection economics should be the development of new levels of analyses in the field of organization and economics of farms (e.g. in terms of strategic and organizational as well as behavioural approach). The empirical conducted research shows the necessity of changes in the policy area and real practices in the examined area (Piwowar 2018a). The purpose of the review of active substances of plant protection products carried out in the European Union for several years is to ensure the safety of consumers and the natural environment (Boucaud-Maitre et al. 2019). A lot of new preparations are being introduced to the market with increasingly high-quality requirements (toxicological and ecotoxicology). A lot of substances were withdrawn from use, which largely limited the choice of agricultural producers. In the case of small farms, the effectiveness of protection using agrotechnical and biological methods can be relatively high. In the case of large farms, the need for stronger motivation to change behaviour in the area of reducing pesticide use is evident. In addition to formal changes, including economic and fiscal stimuli, institutional changes are necessary. It is important to support the creation and development of agricultural institutions and to build social capital. It is necessary to develop professional consultancy in the field of plant protection. Greater awareness among farmers in the field of environmental protection and the need to take into consideration issues regarding pesticide resistance should accelerate the development of the biopesticide market. It is justified, due to the occurrence of negative externalities, to develop a modification of the system that internalizes external costs in the studied issues.

The results of analyses can be used by scientists and decision-makers to assess the existing solutions in the field of pesticide management practices and their contribution to the sustainable development of agriculture. The presented research results can be the basis for comparisons on local, regional and wider spatial scales. Further research directions should also enable interdependence with other factors. Additional variables may take into consideration, inter alia, the state of the environment, the number of incidents related to poisoning people with pesticides and the number of incidents related to bee poisoning. Further studies in this field should also consider other inputs, such as fertilizers. Just like irrational use of pesticides, irrational use of fertilizers can lead to threats to the natural environment. In the case of fertilizers, this applies especially to two components—nitrogen and phosphorus. These nutrients are key in the eutrophication process (Dodds and Smith 2016; Dou et al. 2019). It is worth emphasizing that the consumption of mineral fertilizers in Poland is one of the highest in Europe (Piwowar 2018b; Piwowar 2019).

An important element contributing to sustainable pesticide management is progress and new concepts in precision farming. The key element in this area is the application of an optimal, variable dose of pesticides based on application maps. Practical use of precision farming tools and techniques in the field of pesticides allows reducing environmental and health risks. A model of precision agriculture should be systematically implemented, in which the treatments of precise application of plant protection products are of particular importance. These solutions significantly improve the operator’s safety and reduce the risk of contamination of the natural environment. As technical progress is rapid and the costs of advanced electronic systems in the field of precision farming decrease, these techniques can be implemented not only in large-scale farms. Personal protective equipment also plays a very important role in the area of broadly understood safety and striving for sustainable development in the field of pesticide management (Garrigou et al. 2020). Further research is needed not only in the area of modern technical means for pesticide application and effective personal protective equipment, but also in methods and techniques of pesticide monitoring in ecosystems (analyses of direct and indirect effects of pesticide use on the food chain, interactions of pesticides with other natural and anthropogenic factors, etc.).

The current policy of the European Union aims to reduce the use of pesticides. The European Commission proposes that by 2030, EU countries should reduce the amount of used pesticides by at least 50%. In this way, the European Union plans to stop the problem of the mass extinction of wild pollinators and protect biodiversity. On the other hand, reducing the amount of pesticides used in crops may mean a significant drop in yield, which will consequently lower farm incomes. Therefore, comprehensive changes and an integrated approach to agricultural and environmental policies are necessary (Möhring et al. 2020a, 2020b).

## Summary

In the European Union countries, integrated pest management is an obligation, and in relation to Poland, this requirement has been in force since 2014. Despite the implemented directives, there was no decrease in the use of pesticides in Poland, and moreover, until 2017, there was a systematic increase in the volume of their use. Thus, further actions are necessary to promote agriculture that is more environmentally friendly, including the use of non-chemical methods of plant protection. There is a need to continue control programs for pesticide residues in all agricultural products, especially those where high doses of pesticides are applied (mainly in horticulture).

The main trend of the considerations proposed in the paper is the existence of a relationship between the direction of pesticide consumption (increase, no change, decrease) and the production and economic attributes of farms. Statistical analysis showed the existence of strong relationships between the economic size of farms and the direction of changes in pesticide consumption (*φ*^2^ = 0.1141). Detailed results also make it possible to state the relationship between the direction of changes in pesticide consumption and the size of a farm and its location. The result from multiple correspondence analysis shows that the decrease in pesticide consumption concerned especially small farms, economically weak. These observations can provide useful tips in the real and regulatory sphere. There is insufficient motivation for farmers running large farms to reduce the use of pesticides. The legal provisions lead to both a reduction in the diversity of plant protection products used and to a small extent contribute to the reduction of pesticide use in Polish agriculture. The results of the survey showed the need to increase the knowledge and skills of farmers in the research area. A significant part of the respondents did not know the methodology of IPM, and the cultivation of which was carried out on their farms. In addition to formal changes, including economic and fiscal stimuli, institutional and behavioural changes in farmers are necessary. The growing concentration and specialization of production on farms in Poland is a factor contributing to the strengthening of the role of advisory services in this area. It is the basis for creating coherent strategies and procedures in the area of IPM at the farm level. It is necessary to support further activities in the field of protection and rational use of pesticides in Polish agriculture.
