# Correction to: The use of pesticides in Polish agriculture after integrated pest management (IPM) implementation

**DOI:** 10.1007/s11356-021-12879-w

**Published:** 2021-02-24

**Authors:** Arkadiusz Piwowar

**Affiliations:** Wroclaw University of Economics and Business, Komandorska Street 118/120, 53-345 Wrocław, Poland

**Correction to: Environmental Science and Pollution Research**

10.1007/s11356-020-12283-w

The y-axis legend of Figure 6 is cut-off in the original published paper.

The original article has been corrected.

